# Low Handgrip strength and its lifestyle and physiological correlates among Taiwanese University Students: A cross-sectional study

**DOI:** 10.1371/journal.pone.0350147

**Published:** 2026-06-11

**Authors:** Shao-Yun Chien, Yu-Kuei Teng, Hsiao-Wen Hung, Tsuei-Hung Wang, Mei-Chen Ho, Ya-Ling Tzeng

**Affiliations:** 1 School of Nursing, College of Health Care, China Medical University, Taichung, Taiwan; 2 Department of Nursing, China Medical University Hospital, Taichung,‌‌ Taiwan; 3 Department of Healthcare Administration, Asia University, Taichung, ‌‌Taiwan; 4 PhD Program for Health Science and Industry, College of Health Care, China Medical University, Taichung, Taiwan; Public Library of Science, UNITED KINGDOM OF GREAT BRITAIN AND NORTHERN IRELAND

## Abstract

Handgrip strength is a well-established indicator of muscular fitness and an early predictor of adverse long-term health outcomes. However, limited research has examined HGS and its determinants among young adults in East Asia. This cross-sectional study investigated the association between lifestyle and physiological factors and low HGS in 501 Taiwanese university students aged 18–25 (168 men, 333 women). Participants completed questionnaires on physical activity, sedentary time, and dietary behavior, and underwent anthropometric measurements including body mass index (BMI), mid-upper arm circumference, and handgrip strength. Low handgrip strength was defined using the sex-specific criteria established by the Asian Sarcopenia Working Group in 2019 (men < 28 kg; women <18 kg). Results showed that 6.2% of participants had low handgrip strength (8.3% in men, 5.1% in women). Men had significantly higher average handgrip strength than women (38.0 ± 7.8 kg vs. 24.6 ± 4.4 kg, *p* < .001). Upper arm circumference and handgrip strength showed the strongest correlation in both sexes (*p* < .001). Logistic regression analysis revealed that smaller upper arm circumference (OR = 0.80, *p* = .003 per 1 cm increase), insufficient moderate-to-vigorous physical activity (OR = 0.40, *p* = .055), and male sex (OR = 5.28, *p* = .001) were significantly associated with poor handgrip strength. Dietary habits and sedentary time were not significantly associated with handgrip strength. These findings highlight the importance of early identification of muscle weakness and promoting physical fitness training in young adults. Men with smaller arm circumference and lower activity levels showed a significant association with low HGS and may benefit from early monitoring and targeted intervention. Incorporating muscle strength assessment and activity promotion into early adulthood health programs may help preserve muscular fitness, prevent future sarcopenia, and support healthier aging trajectories in East Asian populations.

## Introduction

Handgrip strength (HGS) is widely recognized as a standard measure for assessing human muscle strength and functional capacity [1]. Its widespread use in clinical and public health domains stems from a straightforward measurement method and exceptional reliability [2,3]. A growing corpus of evidence elucidates that HGS functions as a significant indicator of overall health status and exhibits substantial correlations with the prevalence of chronic diseases, functional decline, and heightened mortality risk [4–6]. Recently, Vaishya et al. (2024) proposed a novel conceptualization of HGS as a new “vital sign,” positing its capacity to reflect musculoskeletal health, identify early disease risk, and monitor functional deterioration [7].

Current research on HGS has predominantly focused on older adults [8]. However, recent studies have demonstrated that even among healthy university students, reduced HGS may be associated with elevated cardiometabolic risk factors [9]. According to the life course model, muscle strength and mass peak between 20 and 30 years of age and then gradually decline [5,10]. Given this pattern, optimizing peak muscle strength in early adulthood is critical to mitigating the functional decline in later life. Research also suggests low muscle strength during adolescence may have long-term health consequences. For instance, a longitudinal study encompassing more than one million Swedish male adolescents revealed that each standard deviation elevation in HGS at the age of 18 correlated with an 11% diminished risk of developing coronary heart disease and a 9% reduced risk of experiencing a stroke in later adulthood [11].

While males typically exhibit higher handgrip strength owing to physiological factors [12,13], recent findings show a gradual decline in their muscle strength levels, possibly driven by modern lifestyle changes [14,15]. Similarly, cultural emphasis on thinness among East Asian women [16] may negatively influence muscle health, highlighting an area that warrants further study. Although sex differences in handgrip strength have been explored in previous research, the physiological and behavioral mechanisms underlying these disparities remain insufficiently understood. As such, conducting sex-stratified analyses when examining factors associated with grip strength is essential for identifying potential determinants.

Regarding anthropometric indicators related to grip strength, mid-upper arm circumference (MUAC) and body mass index (BMI) are commonly used and practically valuable measures. Multiple studies have reported a consistent positive correlation between MUAC and grip strength, whereas the relationship between BMI and grip strength tends to be moderate [17,18]. However, some research has shown that in particular subgroups of younger individuals, BMI is not significantly associated with grip strength [19,20]. In addition, lifestyle behaviors such as insufficient physical activity and prolonged sedentary time may adversely affect muscle function. On the other hand, healthy dietary behaviors are considered potential factors that support overall physical fitness and muscle function. Healthy eating patterns, such as regular meal consumption, increased intake of fruits and vegetables, and avoiding high-fat and high-sugar foods, may help maintain proper nutritional status, thereby promoting muscle strength and overall physical function [21].

University is considered a critical stage for lifestyle transitions. After enrollment, students often break away from established family routines and experience major changes in eating habits, physical activity, and other health behaviors [22]. These changes may have potential impacts on muscle health and grip strength performance.

Although HGS is widely recognized as an indicator of overall health, evidence among healthy young adults—particularly within East Asian populations—remains scarce. Existing research has predominantly focused on older or Western cohorts, providing limited understanding of the early determinants of muscle strength across different cultural and physiological contexts. Furthermore, few studies have simultaneously examined behavioral factors (e.g., physical activity, sedentary behavior, diet) and physiological indicators (e.g., BMI, mid-upper arm circumference) or applied sex-stratified analyses to disentangle biological and sociocultural influences [18,23]. Addressing these gaps, the present study investigates lifestyle and physiological correlates of low HGS among Taiwanese university students, with analyses stratified by sex. By focusing on this transitional life stage, the study aims to provide early evidence for muscle-health surveillance and highlight the importance of strengthening interventions during young adulthood, when preventive action may have the greatest long-term benefit.

## Methods

### Study design

This study was approved by the Institutional Review Board of China Medical University Hospital (approval number: CMUH112-REC1–061). All participants provided written informed consent after being informed of the purpose and procedures of the study. This cross-sectional study was designed to investigate factors associated with HGS among full-time undergraduate students at a medical university in central Taiwan. The central hypothesis of the study was that various sociodemographic, lifestyle, and nutritional factors would influence handgrip strength (HGS) in a young adult population. Specifically, the objective was to examine the relationships between HGS and variables such as body mass index (BMI), mid-upper arm circumference (MUAC), dietary behaviors, average daily sedentary time, and minutes of moderate to vigorous physical activity (MVPA). The independent variables included participant characteristics (age, sex, year of study, living arrangements, commuting mode, and smartphone usage), lifestyle behaviors (sedentary time, MVPA, and dietary habits), and anthropometric measures (BMI and MUAC). The independent variables selected included participant characteristics (age, sex, year of study, living arrangements, commuting mode, smartphone usage), lifestyle behaviors (sedentary time, MVPA, dietary habits), and anthropometric measures (BMI, MUAC). HGS served as the primary dependent variable, chosen for its well-established role as an indicator of overall muscle strength and a potential early marker of health status even in young adults. This design allows for the identification of potential correlations and associations, providing insights into factors that may contribute to HGS variability within this specific student demographic. The study’s cross-sectional nature enables a snapshot of these relationships at a given time.

### Participants

The study was approved by the institutional review board (IRB) of the participating university (IRB protocol number blinded for review). Prior to participation, researchers provided detailed explanations regarding the study’s purpose, procedures, and participant rights. All participants provided appropriate informed consent by signing a consent form. Eligibility criteria for subject selection included full-time undergraduate students at a medical university in central Taiwan, aged 18–25 years. Students were excluded if they had any chronic disease or a history of upper-limb injuries, fractures, surgery, deformities, or musculoskeletal conditions (e.g., joint pain, osteoarthritis, rheumatoid arthritis) that might influence grip-strength assessment. Participants were recruited through open campus announcements, with recruitment strategies accounting for the gender distribution of the university’s overall student population to prevent disproportionate representation of any one sex, enhancing sample representativeness and diversity. All participants’ characteristics such as age, height, and body mass were collected. Participants were prospectively recruited from November 2 to November 30, 2023 through open campus announcements.

### Data collection procedures

Data were collected through a combination of objective physiological measurements and self-administered questionnaires during a single assessment session for each participant. Research staff, trained rigorously prior to data collection, conducted assessments of weight, height, MUAC, and HGS using standardized protocols. Participants also completed structured questionnaires.

HGS was measured using a Jamar hydraulic hand dynamometer (Rolyan, UK), following the standardized protocol of the American Society of Hand Therapists (ASHT) [24,25]. Participants were seated with feet flat on the floor, shoulders adducted and neutrally rotated, elbows flexed at 90 degrees, forearms and wrists in neutral position [26,27]. Each hand was tested alternately three times, with a 30-second rest between trials. The maximum of the three trials for each hand was recorded in kilograms (kg). The Jamar dynamometer used in this study has demonstrated excellent concurrent validity against standardized weights (r = 0.9998; r > 0.96 reported) and is considered the gold standard for grip strength measurement [27]. Due to the cross-sectional design and single testing period for HGS, test-retest reliability coefficients for HGS measurement from our laboratory are not applicable for this specific study design. However, the use of a gold standard instrument and strict adherence to established protocols minimize measurement variability.

Nutritional status was evaluated using two anthropometric indicators: BMI and MUAC. We measured standing height and body weight using a calibrated Seca 709 stadiometer (±0.5 cm) and an Omron HBF-362 scale (±0.1 kg), following standardized procedures. Participants were asked to stand barefoot with their heels together, arms at their sides, and their head positioned in the Frankfort horizontal plane. Height was recorded from the floor to the top of the head by using a right-angle triangle or a flat object placed perpendicular to the stadiometer. Body mass index (BMI) was calculated as weight in kilograms divided by height in meters squared (kg/m²). BMI is a widely used indicator of general body composition and has demonstrated high reliability in repeated anthropometric assessments [28]. According to the World Health Organization’s (WHO) recommendations for Asian populations, individuals with a BMI below 18.5 kg/m² are classified as underweight, 18.5–22.9 kg/m² as normal weight, 23.0–24.9 kg/m² as increased risk, and ≥25.0 kg/m² as obese [29].

A non-elastic Seca 201 measuring tape was used for MUAC. MUAC is widely used as a screening tool for undernutrition. Due to physiological differences in body composition, sex-specific reference values are recommended: a MUAC in the range of 23.5–24.0 cm in males and 21.8–22.0 cm in females is generally considered indicative of undernutrition [30–32].

The Participant Characteristics and Lifestyle Behavior Questionnaire gathered information on age, sex, year of study, current living arrangements, commuting mode to campus, average daily smartphone usage, and sedentary time. Physical activity was assessed using a single-item screening question based on the World Health Organization (WHO) guidelines [33]. Participants were asked whether they engaged in structured, intentional exercise (e.g., running, cycling, sports) for at least 150 minutes per week, sustained for at least six months [33–35]. Consistent with our focus on deliberate exercise, casual walking and household chores were excluded, as they primarily reflect habitual daily movements rather than structured, health-promoting exercise [36]. This approach focuses on ‘structured exercise’ rather than total physical activity, a method previously validated in studies investigating the health effects of deliberate exercise behaviors [37]. These variables were selected to investigate sociodemographic and behavioral factors potentially associated with HGS. Dietary habits were assessed using a 12-item self-administered questionnaire adapted based on dietary recommendations from the Health Promotion Administration, Ministry of Health and Welfare (Taiwan). The questionnaire evaluates key eating behaviors over the past seven days, including fruit and vegetable intake, beverage choices, and meal regularity. Each item is rated on a 4-point Likert scale ranging from 0 (rarely or never) to 3 (daily), yielding a total score between 0 and 36, with higher scores indicating healthier dietary behavior. Total scores are categorized into four levels: 0–12 (very unhealthy), 13–20 (fair), 21–30 (good), and 31–36 (very healthy). While formal psychometric statistics for this government-developed instrument have not been published, the questionnaire has demonstrated content applicability through repeated academic use. It has been applied in multiple peer-reviewed studies across different populations in Taiwan, including investigations of adult dietary behavior, lifestyle surveillance, and clinical dietary interventions [38–40]. In the present sample, the internal consistency was 0.617 (Cronbach’s α), reflecting moderate internal consistency of the scale in the current sample.

The full reliability analysis output and the questionnaire item list are provided in S1 File and S2 File.

### Statistical analyses

All statistical analyses were performed using SPSS for Windows version 25.0 (IBM Corp., Armonk, NY, USA). Descriptive statistics were used to summarize the characteristics of the participants, with continuous variables presented as the mean ± standard deviation (SD) and compared between genders using independent-samples t-tests. Categorical variables were expressed as frequencies and percentages and analyzed using chi-square (χ²) tests. Pearson correlation analysis was conducted to examine the relationships among HGS, BMI, dietary score, MUAC, sedentary time, and MVPA, with variables stratified by sex. Bivariate analyses were performed to explore active patterns related to gender differences in HGS, nutritional status, dietary behavior, and MVPA. Furthermore, logistic regression analyses were conducted to identify factors associated with low HGS (defined as less than 28 kg for males and less than 18 kg for females, according to the Asian Working Group for Sarcopenia (AWGS) 2019 criteria [41]. A multivariate logistic regression model was constructed using the backward elimination procedure to retain only significant variables. Odds ratios (OR), 95% confidence intervals (CI), and p-values were reported for both crude and adjusted models. All statistical tests were two-tailed, and the alpha level for significance was set at ρ ≤ 0.05.

Sample size estimation was conducted using G*Power (version 3.1.9.7) for logistic regression (α = 0.05, power = 0.80). Based on collegiate sarcopenia prevalence [42], 4.2% in females (null probability = 0.042) and 0.5% in males, we assumed an odds ratio (OR) of 0.11. This yielded a required sample of 453; accounting for 10% attrition, we recruited 504 participants. he widely recognized reliability and validity of the Jamar dynamometer and standard anthropometric measurements support the robustness of the data collected. Effect sizes and confidence intervals are reported for odds ratios in the logistic regression analyses.

## Results

### Participant characteristics

A total of 501 university students participated in the study, consisting of 168 males (33.5%) and 333 females (66.5%), with a comparable mean age between sexes (males: 20.16 ± 1.58 years; females: 20.10 ± 1.53 years; *p* = .66). Detailed demographics and basic attribute information are summarized in Table 1. Low HGS was identified in 31 students (6.2% overall), with prevalences of 8.3% in males (14/168) and 5.1% in females (17/333); this sex difference was not statistically significant (*p* = .16). Participants were distributed across all academic years, with no significant sex-based differences in year level (*p* = .09). Most students lived in rented housing, shared housing, or dormitories, with no significant differences in current living arrangements between sexes (*p* = .65).

**Table 1:** Demographics and Basic Attributes by Sex.

Variable	Male (n = 168) n (%)	Female (n = 333) n (%)	p-value
**Low grip strength**			0.16
Yes	14 (8.3)	17(5.1)	
No	154(91.7)	316(94.9)	
**Grade**			0.09
Year 1 (%)	44 (26.2)	88 (26.4)	
Year 2 (%)	25 (14.9)	76 (22.8)	
Year 3 (%)	59 (35.1)	87 (26.1)	
Year 4 (%)	40 (23.8)	82 (24.6)	
**Residence**			0.65
Living with Family	23(13.7)	52(15.6)	
Living in rented housing	59(35.1)	126(37.8)	
Shared Housing	41(24.4)	67(20.1)	
Dormitory	45(26.8)	86(25.8)	
**Commuting**			<0.001
Private Vehicle	120 (73.2)	175 (53.8)	
Walking/Cycling	35 (21.3)	94 (28.9)	
Public Transit	9 (5.5)	56 (17.2)	
≥25.0 kg/m²	32(19)	31(9.3)	
**Dietary Score**			
0–12	24(14.5)	47(14.3)	0.91
13–20	98(59.4)	186(56.5)	
21–30	41(24.8)	92(28.0)	
31–36	2(1.2)	2(1.2)	
	M ± SD	M ± SD	
**Age (years)**	20.16 ± 1.58	20.10 ± 1.53	0.66
**Smartphone Usage** (hrs/day)	6.2 ± 2.5	6.1 ± 2.6	0.52

In terms of commuting patterns, males were more likely to commute by private vehicle, while females reported higher rates of walking, cycling, or using public transportation (*p* < .001). Daily smartphone usage was similar between groups (males: 6.2 ± 2.5 hr/day; females: 6.1 ± 2.6 hr/day; *p* = .52). There was no significant difference in dietary scores between male and female students (*p* = .91). More than half of the students exhibited average dietary habits (males: 59.4%; females: 56.5%); approximately 14% in each group reported very unhealthy eating patterns, and only 1.2% of students in both groups reported very healthy diets.

Significant sex-based differences were observed in sedentary time and physical activity levels. Female students reported longer average sedentary time than males (9.3 ± 3.0 vs. 8.0 ± 2.7 hr/day; *p* < .001), and a higher proportion of males reported engaging in ≥150 minutes of MVPA per week (63.7% vs. 24.2%; *p* < .001). Regarding BMI, a greater proportion of females were underweight (BMI < 18.5 kg/m²: 16.5% vs. 6.0%), while more males were classified as overweight or obese (BMI ≥ 25.0 kg/m²: 19.0% vs. 9.3%; *p* < .001).

### Sex differences in handgrip strength, nutritional status, and lifestyle behaviors

As shown in Table 2, male participants demonstrated significantly higher HGS (38.0 ± 7.8 kg) compared to females (24.6 ± 4.4 kg; p <.001). Males also had significantly higher BMI (22.4 ± 3.3 vs. 21.2 ± 3.2 kg/m²; p <.001), and greater MUAC (31.7 ± 3.8 cm vs. 27.4 ± 3.0 cm; p <.001). In contrast, dietary behavior scores did not differ significantly between sexes (male: 17.6 ± 4.9; female: 17.8 ± 5.0; p =  .65), suggesting similar dietary habits among male and female students. Significant sex-based differences were observed in sedentary and regular MVPA time. Additionally, female students reported longer average sedentary time than males (9.3 ± 3.0 vs. 8.0 ± 2.7 hr/day; p <.001), and a higher proportion of males reported engaging in ≥150 minutes of MVPA per week (63.7% vs. 24.2%; p <.001).

**Table 2:** Comparison of Handgrip Strength, Nutritional Indicators, and Physical Activity by Sex.

Variable	Male (n = 168) M ± SD	Female (n = 333) M ± SD	p-value
Handgrip Strength (kg)	38.0 ± 7.8	24.6 ± 4.4	<0.001
BMI (kg/m²)	22.4 ± 3.3	21.2 ± 3.2	<0.001
MUAC (cm)	31.7 ± 3.8	27.4 ± 3.0	<0.001
Dietary behavior	17.6 ± 4.9	17.8 ± 5.0	0.65
Sedentary (hr/day)	8.0 ± 2.7	9.3 ± 3.0	<0.001
	n (%)	n (%)	
MVPA ≧150 min/week			<0.001
Yes	107 (63.7%)	78 (24.2%)	
No	61 (36.3%)	244 (75.8%)	

### Correlation between handgrip strength and health indicators by sex

**Fig 1:**
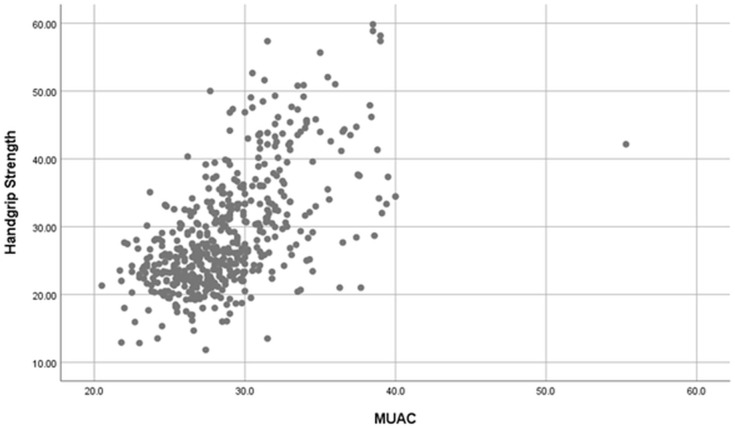
Association between mid-upper arm circumference and handgrip strength. The scatter plot shows the relationship between mid-upper arm circumference (MUAC) and handgrip strength (HGS) ‌‌among participants. MUAC, mid-upper arm circumference; HGS, handgrip strength.

Table 3 shows that in the overall sample, HGS was weakly but significantly negatively associated with sedentary time (r = –0.170, *p* < .001), and positively associated with BMI (r = 0.301, *p* < .001) and MUAC (r = 0.713, *p* < .001). No significant association was found with dietary score (r = 0.031, *p* = .497). When stratified by sex, MUAC demonstrated the strongest and most consistent correlations with HGS in both males (r = 0.424, *p* < .001) and females (r = 0.854, *p* < .001). As shown in Fig 1, the scatter plot illustrates the strong positive association between MUAC and handgrip strength. BMI also showed moderate positive correlations with HGS in both males (r = 0.233, *p* < .002) and females (r = 0.310, *p* < .001). Sedentary time was negatively correlated with HGS in males (r = –0.110), though not statistically significant (*p* = .155), and showed no meaningful association in females (r = 0.039, *p* = .475). Dietary score remained unrelated to HGS in both sexes.

**Table 3:** Pearson Correlation Coefficients Between Handgrip Strength and Health Indicators, Stratified by Sex.

Variable	Group	n	r	p-value
Sedentary Time vs. HGS	Male	168	–0.110	0.155
	Female	332	+0.039	0.475
	Total	500	–0.170	<.001
BMI vs. HGS	Male	168	0.233	<0.002
	Female	333	0.310	<0.001
	Total	501	0.301	<0.001
MUAC vs. HGS	Male	168	0.424	< 0.001
	Female	333	0.854	<0.001
	Total	501	0.713	<0.001
Dietary Score vs. HGS	Male	165	0.067	0.391
	Female	329	0.019	0.733
	Total	494	0.031	0.497

Note: The sample size for each analysis varies slightly due to missing data in some variables.

### Logistic regression model including lifestyle and physiological factors

A stepwise logistic regression model was constructed to evaluate the association between HGS and a broader set of factors, including MVPA, gender, commuting method, BMI category, average sedentary time, and MUAC. The final model retained three significant factors: MVPA, gender, and MUAC (see Table 4). Compared to students who engaged in <150 minutes of MVPA per week, those who exercised ≥150 minutes were less likely to exhibit low risk for low handgrip strength (OR = 0.395, 95% CI: 0.153–1.022, *p* = .055). Male sex has a higher risk of low handgrip strength compared to females, with an odds ratio of 5.275 (OR = 5.275, 95% CI: 1.982–14.040, *p* = .001). Arm circumference was negatively associated with the likelihood of low grip strength (OR = 0.80, 95% CI: 0.691–0.926, *p* = .003), indicating that for each 1 cm increase in arm circumference, the odds of low grip strength decreased by approximately 20%. Greater arm circumference was associated with lower odds of low grip strength (OR = 0.80, *p* = .003). Other variables initially considered in the model, including commuting method, BMI, and sedentary time, were excluded in the final step due to lack of statistical significance.

**Table 4:** Logistic regression to identify significant factors of low handgrip strength.

Variable (Reference)	B	SE	Wald χ²	p-value	aOR	95% CI
MVPA (<150 min/week)	−0.928	0.484	3.669	0.055	0.395	0.153–1.022
Gender (Females)	1.663	0.500	11.083	0.001	5.275	1.982–14.040
Arm Circumference (cm)	−0.223	0.074	8.993	0.003	0.800	0.691–0.926

Note. aOR = adjusted odds ratio; CI = confidence interval.

### Sensitivity analysis

To evaluate the robustness of our findings, a sensitivity analysis was performed by treating MVPA as ordinal categories (<150, 150–180, and >180 min/week). The results (S1 Table) showed that the associations remained consistent with the main analysis. MVPA remained non-significant (*p* = 0.208), while gender (aOR = 5.84, *p* < 0.001) and mid-upper arm circumference (aOR = 0.79, *p* = 0.001) remained significant independent predictors of low handgrip strength.

## Discussion

This study assessed 501 Taiwanese university students (168 males and 333 females) based on the HGS cut-off values proposed by the Asian Working Group for Sarcopenia 2019 (AWGS-2019) [41]. This criterion is less frequently applied to university-aged populations. Low HGS was identified in 31 participants (6.2% overall), including 8.3% of males and 5.1% of females. Three independent factors associated with low strength were identified: insufficient MVPA (<150 min/week, doubling the risk), smaller MUAC (each additional centimeter associated with an estimated 20% reduction in risk), and male sex (five-fold higher adjusted risk).These findings highlight physical activity and regional muscularity as modifiable factors for early intervention and affirm sex as a key covariate despite modest prevalence differences. This study fills an evidence gap regarding muscle strength in university-aged populations and identifies actionable targets for prevention.

Notably, although the proportion classified as having low HGS was relatively small under the AWGS-2019 criteria, the 25th percentile average HGS in this sample (31.95 kg in males and 21.7 kg in females) exceeded the respective cut-off points. This suggests that the true prevalence of low HGS, if defined by sample-based percentiles, would be significantly higher. This highlights the challenge of applying geriatric-derived screening criteria to younger adult populations, where normative strength levels are substantially higher. Moreover, the fact that some male students were classified as low strength despite relatively high absolute values suggests a possible trend of declining muscular fitness among younger males. This phenomenon has been reported in prior studies, indicating a secular decline in male grip strength over recent decades, possibly due to increased sedentary behavior and reduced physical demands in daily life [14,15]. Although males generally exhibited higher absolute HGS values than females, logistic regression identified male sex as a factor associated with low HGS when using the AWGS-2019 sex-specific cut-off points (<28 kg for males; < 18 kg for females). This finding reflects the relative proportion of males who did not reach their sex-specific reference threshold rather than an overall lower muscle strength compared to females. In other words, while average male grip strength is higher, the criterion-adjusted definition of “low HGS” classifies a subset of males as below standard, resulting in an odds ratio greater than 1. This interpretation aligns with previous findings suggesting that normative thresholds derived from older adult populations may not optimally represent younger cohorts. Such findings underscore the importance of sex-stratified analysis and the need to establish age-appropriate normative grip strength references for early identification and prevention strategies.

In this study, male students had significantly higher HGS than female counterparts, which is consistent with previous literature [43,44]. This sex difference can be primarily attributed to physiological and anatomical characteristics, including greater skeletal muscle mass, upper limb muscle cross-sectional area, and larger forearm circumference and hand width in males [12,45]. In the present sample, male participants also reported higher levels of physical activity, greater BMI, and larger MUAC, further supporting the critical role of physiological factors in the development of muscular strength. Beyond biological determinants, sociocultural and psychosocial influences may also contribute to sex-based disparities in muscular strength. In East Asian societies, such as Taiwan, women often face societal pressure to maintain a thin body shape, resulting in lower participation in resistance training and physical activity in general [46,47]. According to national health statistics, 15.5% of Taiwanese women aged 18–24 are classified as underweight [21], which may further constrain muscle development and functional performance. Many young women aiming for an ideal body weight tend to have reduced lean body mass (LBM) [48,49], and underweight females often exhibit lower dietary diversity and insufficient energy and protein intake, conditions linked to decreased muscular strength and increased risks of malnutrition [50,51]. Additionally, dietary patterns common in East Asian countries, such as a higher reliance on carbohydrates and lower protein intake, may also play a role in muscle development and functional strength. In Taiwan, diets are typically high in carbohydrates—mainly from refined staples such as rice and noodles, while sources of animal protein are comparatively limited [52,53].

In contrast, male students in East Asia may experience less pressure related to body image ideals but often face strong sociocultural expectations emphasizing academic achievement over physical fitness [54]. Such academic-oriented cultural values may overshadow the importance of exercise and contribute to lower physical activity levels among some male students. Moreover, research suggests that young East Asian men perceive body image through culturally specific frameworks, where the interaction between physique ideals and academic performance is influenced by broader social norms and cultural values [55]. These findings emphasize the need for culturally appropriate interventions that promote both physical activity and healthy body image across genders in East Asia.

In comparison, the average HGS of Taiwanese university students is generally lower than that of their peers in Western countries. This may be influenced by multiple factors, including interethnic genetic differences, physical education systems, fitness culture, nutritional intake, and body composition [56,57]. According to a United Nations Educational, Scientific and Cultural Organization (UNESCO) survey, East Asian countries typically allocate less time to school-based physical education compared to Western nations [58]. In addition, heavier academic workloads and limited access to exercise resources may adversely affect physical development. However, even within Asia, the HGS of these Taiwanese male students is lower than that of their Japanese and Thai counterparts, while the grip strength of female students is slightly higher than that of Korean females. This variation may be related to prevailing cultural values in Taiwan and South Korea, where thin body ideals are widely promoted in media [46,47], potentially influencing body-related behaviors and body composition among younger generations. Overall, the results of this study emphasize the importance of early exercise intervention and health promotion for young people. Future research should further explore the impact of gender and cultural factors on grip strength development.

Among the various indicators examined, MUAC demonstrated the strongest and most consistent association with HGS, particularly in females (r = 0.854, *p* < .001), and to a lesser extent in males (r = 0.424, *p* < .001). This highlights the importance of upper-arm muscle mass as a determinant of functional strength in university students, as also supported by previous studies [17,59]. BMI also showed moderate positive correlations with HGS in both sexes (female: r = 0.310; male: r = 0.233), which aligns with previous studies indicating that higher BMI, particularly with greater lean mass, may be associated with stronger grip [18]. Sedentary time was negatively correlated with HGS in the total sample (r = –0.170, *p* < .001), yet not statistically significant when stratified by sex. This could suggest subtle sex-specific differences in response to sedentary behavior or insufficient statistical power in subgroup analyses. Interestingly, dietary behavior showed no significant association with HGS (r = 0.031, *p* = .497 overall). This lack of association might reflect limitations of the brief self-reported scoring system, which primarily captures short-term eating patterns rather than long-term dietary habits that may have a stronger influence on muscle strength.

The findings from the logistic regression model highlight the combined influence of weekly MVPA time, sex, and MUAC on HGS in the studied student population. Among these factors, sex exhibited the strongest association, with male students being over five times more likely to have a higher risk of low HGS compared to females (OR = 5.275, *p* = .001). This substantial odds ratio underscores the impact of biological sex on muscular strength vulnerabilities in this cohort, suggesting that certain male subgroups may be particularly susceptible due to factors such as lower physical activity levels or unfavorable body composition [60]. MUAC demonstrated a significant protective association against low HGS (OR = 0.80, *p* = .003). Specifically, for each 1 cm increase in arm circumference, the odds of exhibiting low grip strength decreased by approximately 20%. This result suggests that greater arm girth, potentially reflecting greater muscle mass, serves as a protective factor against strength deficits [12,45]. Weekly MVPA time (≥150 minutes versus <150 minutes) exhibited a borderline significant inverse association with low handgrip strength (OR = 0.395, *p* = .055). Although the p-value slightly exceeded the conventional significance threshold, the trend indicates that higher levels of physical activity might mitigate the risk of diminished HGS. Nevertheless, the borderline significance and absence of detailed classification regarding exercise type and intensity necessitate cautious interpretation and warrant further investigation [61,62].

This study has several notable strengths and limitations. First, it addresses a critical gap in epidemiological data on muscular strength among young adults by applying the updated AWGS-2019 criteria to a university-aged population. Second, the sample size was adequate (N = 501), and both subjective (self-reported lifestyle questionnaires) and objective (HGS and MUAC) indicators were employed. This multimethod approach helps mitigate the risk of common method bias and enables sex-stratified analyses, which enhance the interpretability and relevance of the findings. Importantly, the results support the notion that sex may exert an independent effect on muscular strength, even in contexts with only minimal differences in prevalence. The study also identifies two modifiable factors—MVPA and MUAC—that could serve as practical indicators for early monitoring and intervention, before the onset of age-related declines in muscle function. These findings offer actionable insights for university health services and public health initiatives aimed at the early prevention of sarcopenia. Moreover, grip strength in early adulthood may serve as a valuable predictor of future sarcopenia and functional decline. Research has shown that lower HGS at a young age is associated with an increased risk of developing sarcopenia later in life [63,64]. Early identification of low HGS among university students, therefore, provides an opportunity for preventive strategies to maintain muscle health across the lifespan. These findings offer actionable insights for university health services and public health initiatives aimed at the early prevention of sarcopenia.

However, several limitations should be acknowledged. First, the lifestyle variables—including physical activity, sedentary time, and dietary behaviors—were assessed using self-reported questionnaires, which may be influenced by recall and social desirability bias. In addition, brief self-report dietary measures in young adults may exhibit limited discrimination, which could attenuate associations between dietary behaviors and health outcomes. Furthermore, because our measure of physical activity excluded walking and domestic activities and focused on structured exercise, it likely underestimated total physical activity and may have introduced non-differential misclassification. Nevertheless, similar self-administered instruments have been widely applied and validated in epidemiological studies among college students and young adults [40,65,66], with evidence demonstrating acceptable reliability and validity, as well as the feasibility of simplified measures of physical activity, lifestyle, and dietary behaviors in these populations. Additionally, the brief dietary behavior questionnaire used in this study may not fully capture detailed nutrient intake, such as protein or micronutrients, which are important for muscle strength. However, this simplified tool was selected to ensure feasibility and reduce respondent burden, as longer dietary surveys may lower completion rates among university students. Second, the definition of low grip strength used the AWGS-2019 criteria, which were derived from an older adult population, which may underestimate the actual prevalence of weak grip in young adults. It is thus recommended to establish local normative data for grip strength in young adults to more accurately identify muscle strength deficiencies in this population. Third, this study employed a cross-sectional design, which limits the ability to infer causal relationships between handgrip strength and associated risk factors. To address this limitation, future studies using longitudinal designs may better clarify temporal changes to track temporal changes in handgrip strength and related health outcomes, thereby enhancing the understanding of its role in predicting health risks among young populations. Finally, the study sample was primarily composed of university students from a specific region and educational background, which may limit the generalizability of the findings. Given that lifestyle behaviors, nutritional intake, and physical activity levels can vary substantially across regions, the external validity of the results may be constrained. Furthermore, as all participants were enrolled at a medical university, their health awareness, academic workload, and lifestyle patterns may differ from those of students in non–health-related disciplines, which could further limit the generalizability of the findings. To improve the broader applicability of future findings, it is advisable to include more diverse population samples‌‌ that reflect the heterogeneity of young individuals across different cultural and socio-demographic contexts.

## Conclusions

This study identified male sex, smaller MUAC, and insufficient MVPA as key factors associated with low HGS in a sample of Taiwanese university students. These findings underscore the need for early surveillance and intervention strategies to promote muscular fitness in young adults, particularly targeting at-risk male subgroups. Given the established link between muscular strength and long-term health outcomes, integrating physical activity promotion and muscle mass preservation into early adulthood health initiatives is crucial. Although limitations such as the use of cutoffs derived from older adult populations and self-reported data exist, this study highlights the importance of developing age-appropriate strength norms and advancing longitudinal research to inform effective public health interventions. Promoting muscular strength from early adulthood may serve as a foundational strategy to prevent future functional decline and chronic disease, contributing to healthier aging trajectories at the population level.

## Supporting information

S1 File Reliability analysis output for the 12-item Dietary Behaviour Questionnaire. (XLSX)

S2 File Dietary Behaviour Questionnaire. (DOCX)

S3 File SPSS file used for the statistical analyses. (SAV)

S1 Table Sensitivity analysis using ordinal MVPA categories. (DOCX)

S1 Dataset Anonymized dataset used in the analysis. (XLSX)
