# Supplementary material for: Low Handgrip strength and its lifestyle and physiological correlates among Taiwanese University Students: A cross-sectional study
Source: PLoS One. 2026 Jun 11;21(6):e0350147. doi: 10.1371/journal.pone.0350147 (PMC13257985; doi:10.1371/journal.pone.0350147)
Supplement: S2 File — (DOCX) [file pone.0350147.s002.docx]

**Supplementary Material 2.**

**Diet Behavior Questionnaire (English version translated from the original Chinese instrument)**

| **Number** | **Illustrations** | **Seldom** | **Occasionally** | **Often** | **Always** |
| --- | --- | --- | --- | --- | --- |
| Please select the appropriate answer based on your dietary habits over the past week.  **Always:** ≧8 out of 10 times.  **Often:** 6–8 out of 10 times.  **Occasionally:** 3–5 out of 10 times.  **Seldom:** 1–2 times out of 10 or never. | | | | | |
| 1 | I eat three regular meals a day. | □ | □ | □ | □ |
| 2 | I do not eat sweets or snacks. | □ | □ | □ | □ |
| 3 | I chew food thoroughly (at least 20 times per mouthful). | □ | □ | □ | □ |
| 4 | I drink plain water instead of sugary beverages (soda, sweetened tea/coffee). | □ | □ | □ | □ |
| 5 | I avoid deep-fried or high-oil foods (e.g., cashews, peanuts, potato chips). | □ | □ | □ | □ |
| 6 | I eat fruit every day. | □ | □ | □ | □ |
| 7 | I eat green vegetables every day. | □ | □ | □ | □ |
| 8 | I eat late-night snacks. | □ | □ | □ | □ |
| 9 | I eat while watching TV or reading. | □ | □ | □ | □ |
| 10 | I eat to relieve stress when I am in a bad mood. | □ | □ | □ | □ |
| 11 | I use food as a reward or for celebration. | □ | □ | □ | □ |
| 12 | I only go shopping for food when I am very hungry. | □ | □ | □ | □ |

**Scoring Calculation**

The total score ranges from **0 to 36**.

- **For Items 1–7 (Positive behaviors):** Always = 3, Often = 2, Occasionally = 1, Seldom = 0.
- **For Items 8–12 (Negative behaviors):** Always = 0, Often = 1, Occasionally = 2, Seldom = 3.

**Interpretation / Category Thresholds**

The dietary behavior is categorized into four levels based on the total score:

- **0–12:** Very unhealthy. The dietary behavior is considered unhealthy; weight management or lifestyle intervention is recommended.
- **13–20:** Fair. Specific dietary habits need adjustment.
- **21–30:** Good. The individual has a solid foundation but could improve minor areas.
- **31–36:** Very healthy. The individual has very healthy dietary habits.
