# Supplementary material for: Low Handgrip strength and its lifestyle and physiological correlates among Taiwanese University Students: A cross-sectional study
Source: PLoS One. 2026 Jun 11;21(6):e0350147. doi: 10.1371/journal.pone.0350147 (PMC13257985; doi:10.1371/journal.pone.0350147)
Supplement: S1 Table — (DOCX) [file pone.0350147.s004.docx]

**Supplementary Material 3.**

**Table. Logistic regression analysis of factors associated with low handgrip strength (Sensitivity Analysis with ordinal MVPA categories)**

| Variable (Reference) | B | SE | Wald χ² | p-value | aOR | 95% CI |
| --- | --- | --- | --- | --- | --- | --- |
| MVPA (<150 min/week) |  |  | 3.141 | 0.208 |  |  |
| MVPA (150-180 min/week) | 0.808 | 0.484 | 2.755 | 0.097 | 2.244 | 0.864–5.831 |
| MVPA (>180 min/week) | -0.065 | 1.101 | 0.003 | 0.953 | 0.937 | 0.108–8.107 |
| Gender  (Female) | 1.7764 | 0.495 | 12.691 | <0.001 | 5.837 | 2.211-15.408 |
| Arm Circumference (cm) | -0.242 | 0.075 | 10.500 | 0.001 | 0.785 | 0.678–0.909 |
